# MSC-regulated lncRNA MACC1-AS1 promotes stemness and chemoresistance through fatty acid oxidation in gastric cancer

**DOI:** 10.1038/s41388-019-0747-0

**Published:** 2019-02-11

**Authors:** Wanming He, Bishan Liang, Chunlin Wang, Shaowei Li, Yang Zhao, Qiong Huang, Zexian Liu, Zhiqi Yao, Qijing Wu, Wangjun Liao, Shuyi Zhang, Yajing Liu, Yi Xiang, Jia Liu, Min Shi

**Affiliations:** 1 Department of Oncology, Nanfang Hospital, Southern Medical University, Guangzhou, China; 2 Sun Yat-sen University Cancer Center, State Key Laboratory of Oncology in South China, Collaborative Innovation Center for Cancer Medicine, Guangzhou, Guangdong China

**Keywords:** Gastric cancer, Self-renewal

## Abstract

Chemotherapy is the preferred treatment for advanced stage gastric cancer (GC) patients and chemotherapy resistance is the major obstacle to effective cancer therapy. Increasing evidence suggests that mesenchymal stem cells (MSCs) make important contributions to development of drug resistance. However, the underlying mechanism remains elusive. In this study, we discovered that abundant MSCs in tumor tissues predicted a poor prognosis in GC patients. MSCs promoted stemness and chemoresistance in GC cells through fatty acid oxidation (FAO) in vitro and in vivo. Mechanically, transforming growth factor β1 (TGF-β1) secretion by MSCs activated SMAD2/3 through TGF-β receptors and induced long non-coding RNA (lncRNA) MACC1-AS1 expression in GC cells, which promoted FAO-dependent stemness and chemoresistance through antagonizing miR-145-5p. Moreover, pharmacologic inhibition of FAO with etomoxir (ETX) attenuated MSC-induced FOLFOX regiment resistance in vivo. These results suggest that FAO plays an important role in MSC-mediated stemness and chemotherapy resistance in GC and FAO inhibitors in combination with chemotherapeutic drugs present as a promising strategy to overcome chemoresistance.

## Introduction

Gastric cancer (GC) is a critical health burden and the second common cause of cancer-related death globally [1]. Chemotherapy is the preferred treatment for advanced stage GC patients. 5-Florouracil (5-FU)-based chemotherapy regimens, such as 5-FU in combination with cisplatin (DDP) or oxaliplatin, are generally accepted as the first-line treatments in advanced GC [2–4]. Despite the efficacy of chemotherapy for patients, the survival of patients with GC is substantially worse than that of patients with most other solid malignancies and chemotherapy still achieves a limited response rate [5, 6]. Development of chemoresistance, whether intrinsic or acquired, is a major challenge to efficacy of GC treatment.

The mechanism of GC developing chemotherapy resistance is complicated and not well understood. Both genetic and microenvironmental factors can lead to chemoresistance. As an important part of tumor environment, mesenchymal stem cells (MSCs) play a significant role in the resistance of anticancer drugs [7, 8], wherein the acquisition of increased stemness property in cancer cells is one of the important mechanisms that MSCs confer resistance to chemotherapy [7–9]. However, little is known about the role of metabolic changes of stemness acquisition in cancer cells during MSC-induced chemoresistance.

Cancer cells undergo metabolic reprogramming to support their growth and progression in the complex tumor microenvironment. Fatty acids (FAs) are important energy resources through FA oxidation (FAO), which has been shown to be required for cancer cell growth and survival [10]. Recently, it is reported that FAO is able to support breast cancer stem cell self-renewal and drug resistance but exert little effect on non-stem cancer cells [11]. Inhibiting FAO represses stemness and alleviates tumor growth [10, 12, 13]. Nonetheless, whether FAO is involved in MSC-induced stemness and chemoresistance in GC cells remains unknown.

With the advancement in high-throughput transcriptome analysis by next-generation sequencing in recent years, a large number of long non-coding RNAs (lncRNAs) have been discovered [14]. Increasing evidence has indicated that lncRNAs can participate in multiple tumor biological processes, including maintaining stemness properties and drug resistance [15–17]. We previously reported that lncRNA MACC1-AS1 can facilitate metabolic plasticity through antioxidant production, alleviating with a lower reactive oxygen species (ROS) load under metabolic stress [18]. Lower ROS levels are critical for maintaining stemness and drug resistance [19–21]. Thus we hypothesized that MACC1-AS1 may contribute to stemness and chemoresistance.

Herein we provide evidence that MSCs promote stemness and chemoresistance in GC cells through FAO. Mechanically, transforming growth factor β1 (TGF-β1) secreted by MSCs activated SMAD2/3 through TGF-β receptors, which then induced lncRNA MACC1-AS1 expression in GC cells and promoted FAO-dependent stemness and chemoresistance through antagonizing miR-145-5p.

## Results

### MSCs promote stemness and chemoresistance and predict a poor prognosis in GC

**Fig. 1:**
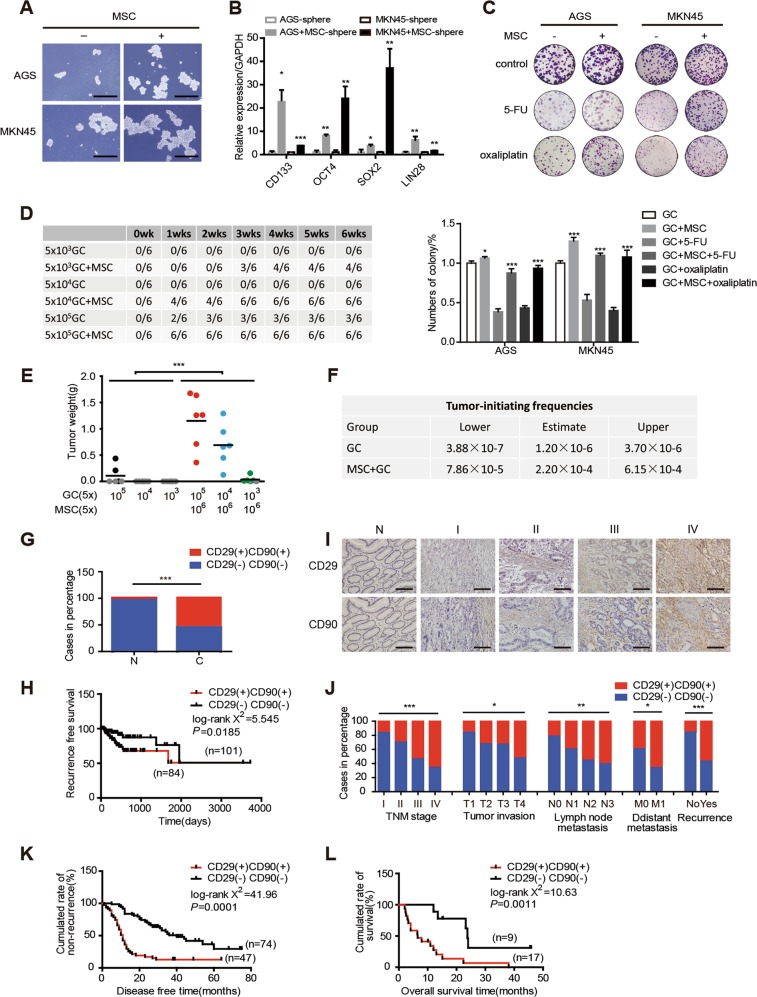
Mesenchymal stem cells (MSCs) promote stemness and chemoresistance and predict a poor prognosis in gastric cancer (GC). **a** Representative images of sphere-formation assay in AGS and MKN45 culture either alone or with MSCs. Scale bar = 500 μm. **b** Expression levels of stemness-associating genes measured by quantitative real-time polymerase chain reaction (qRT-PCR) in AGS and MKN45 sphere co-culture with or without MSCs. **c** Colony-formation assay and the quantitative graph of AGS and MKN45 with or without MSCs when treated with 1 μg/mL 5-florouracil and 3 μg/mL oxaliplatin. **d** Numbers of tumor formation after injection of MKN45 cells with or without MSCs. **e** Tumor weights derived from either different dosages of MKN45 cells or together with MSCs. Gray dots indicate that no tumor grew at the site of injection. Other color dots indicate individual tumor weights. **f** The ranges of estimated tumor-initiating cell frequencies evaluated by ELDA web tool (http://bioinf.wehi.edu.au/software/elda) with 95% confidence. **g** Percentage of double-negative CD29 and CD90 (CD29(−)CD90(−)) and double-positive CD29 and CD90 (CD29(+)CD90(+)) expression in The Cancer Genome Atlas (TCGA) database. **h** Kaplan–Meier curves of postoperative recurrence of stage I–III GC in CD29(+)CD90(+) patients in TCGA database. **i** Representative immunohistochemical staining of CD29 and CD90 in stage I–IV GC and normal gastric tissues. Scale bar = 100 μm. **j** The frequency of CD29(−)CD90(−) and CD29(+)CD90(+) expression in GC categorized by tumor, node metastasis stage (*P* = 0.000464, *χ*^2^ test), tumor invasion (*P* = 0.047, *χ*^2^ test), lymph node metastasis (*P* = 0.004, *χ*^2^ test), distant metastasis (*P* = 0.017, *χ*^2^ test), recurrence (*P* = 0.000002, *χ*^2^ test). **k**–**l** Kaplan–Meier analysis of disease-free survival (stage I–III GC patients) and overall survival (stage IV GC patients) in response to the co-expression of CD29 and CD90. *P < 0.05; **P < 0.01; ***P < 0.001

In order to determine whether MSCs can promote stemness and chemoresistance in GC cells, GC cell lines AGS and MKN45 were co-cultured with MSCs via transwell co-culture system. After co-culturing, stemness genes CD133, OCT4, SOX2 and LIN28 expression levels were enhanced in both mRNA and protein levels (Supplementary Fig. 1a-b). The population of CD44 positive (CD44^+^) GC cells, recognized as cancer stem cell (CSC) typical markers, was significantly increased as detected by flow cytometric analysis (Supplementary Fig. 1c). Moreover, sphere-formation ability of GC cells and the expression levels of stemness genes in sphere-forming GC cells were obviously increased when co-cultured with MSCs indicating the enhanced self-renewal capability of GC cells (Fig. 1a, b). Colony-formation assay showed that growth inhibition effects of 1 μg/mL 5-FU and 3 μg/mL oxaliplatin on GC cells were remarkably decreased in the presence of MSCs (Fig. 1c). These findings suggested that MSCs can promote stemness and chemoresistance in GC cells.

To further investigate the effect of MSCs on tumor initiation, we subcutaneously injected limiting dilution of MKN45 cells at three dosages, 5 × 10^3^, 5 × 10^4^, and 5 × 10^5^, with or without 5 × 10^6^ admixed MSCs. Compared to injection of GC cells alone, the presence of MSCs increased the tumor-initiating frequencies from 0/6, 0/6, and 3/6 to 4/6, 6/6, and 6/6. Notably, injection of GC cells alone was not able to induce tumor formation even with the cell count of 5 × 10^3^ or 5 × 10^4^ cells after 6 weeks. However, when they were mixed with 5 × 10^6^ MSCs, tumor formation occurred within 3 weeks or 1 week, which indicates that MSCs induce de novo tumor formation of GC cells (Fig. 1d, e). As measured by the extreme limiting dilution analysis (ELDA) [22], the frequency of tumor initiation in GC cells alone was from 3.88 × 10^–7^ to 3.70 × 10^–6^; in the presence of admixed MSCs the frequency was increased, from 7.86 × 10^−5^ to 6.15 × 10^−4^ (Fig. 1f). These results indicate that MSCs enhance tumor initiation and are responsible for facilitating de novo tumor formation.

To determine whether MSCs were recruited to tumor tissues and accounted for poor outcome of GC patients, we analyzed the expression levels of MSC surface antigen markers, CD29 and CD90, in The Cancer Genome Atlas (TCGA) database. CD29 and CD90 double-positive MSCs were more abundant in GC tissue as compared to non-cancerous gastric tissue and also associated with shortened recurrence-free survival (RFS) in patients (Fig. 1g, h). Additionally, we analyzed the expression levels of CD29 and CD90 in primary GC samples and matched adjacent nontumorous gastric tissues from 151 patients by immunohistochemistry (IHC). The results showed that the expression levels of CD29 and CD90 were expression correlated with more advanced tumor, node metastasis stages, tumor invasion, lymph node, and distant metastases and recurrence (Fig. 1i, j and Table 1). Kaplan–Meier analysis showed that higher co-expression of CD29 and CD90 was correlated with worse disease-free survival (DFS) (stage I–III GC patients) and overall survival (OS) (stage IV GC patients) (Fig. 1k, l). Additionally, the score of stemness gene OCT4 was higher in CD29 and CD90 double-positive GC tissues in comparison with CD29 and CD90 double-negative GC tissues (Supplementary Fig. 1d-e). These results suggest that MSCs promote stemness and chemoresistance and predict a poor outcome in GC patients.

**Table1:** Relationships between CD29 and CD90 co-expression and clinicopathological parameters in 151 stage I–IV GC patients

	*n* (%)	CD29(−)CD90(−)	CD29(+)CD90(+)	*P* value
		*n*	*n*	
Age (years)				
≥55	93 (63.27)	48	45	0.125
<55	54 (37.73)	35	19	
Gender				
Male	98 (66.67)	56	42	0.861
Female	49 (33.33)	27	22	
TNM stage				
I	25 (17.00)	21	4	0.000464***
II	34 (23.13)	24	10	
III	62 (42.18)	29	33	
IV	26 (17.69)	9	17	
Tumor invasion				
T1	13 (10.65)	11	2	0.047*
T2	19 (15.58)	13	6	
T3	34 (27.87)	23	11	
T4	56 (45.90)	27	29	
Lymph node metastasis				
N0	48 (39.34)	38	10	0.004**
N1	23 (18.86)	14	9	
N2	31 (25.41)	14	17	
N3	20 (16.39)	8	12	
Distant metastasis				
M0	121 (82.31)	74	47	0.017*
M1	26 (17.69)	9	17	
Tumor differentiation				
Well	23 (15.65)	16	7	0.156
Moderate	47 (31.97)	29	18	
Poor	77 (52.38)	38	39	
Recurrence				
No	46 (31.29)	39	7	0.000002***
Yes	101 (68.71)	44	57	

CD29(−)CD90(−) and CD29(+)CD90(+) were determined by total immunohistochemical scores of 0–5 and 6–12, respectively

*GC* gastric cancer, *TNM* tumor, node, metastasis

*P < 0.05, **P < 0.01, ***P < 0.001

### FAO plays an important role in MSC induced-stemness and chemoresistance

**Fig. 2:**
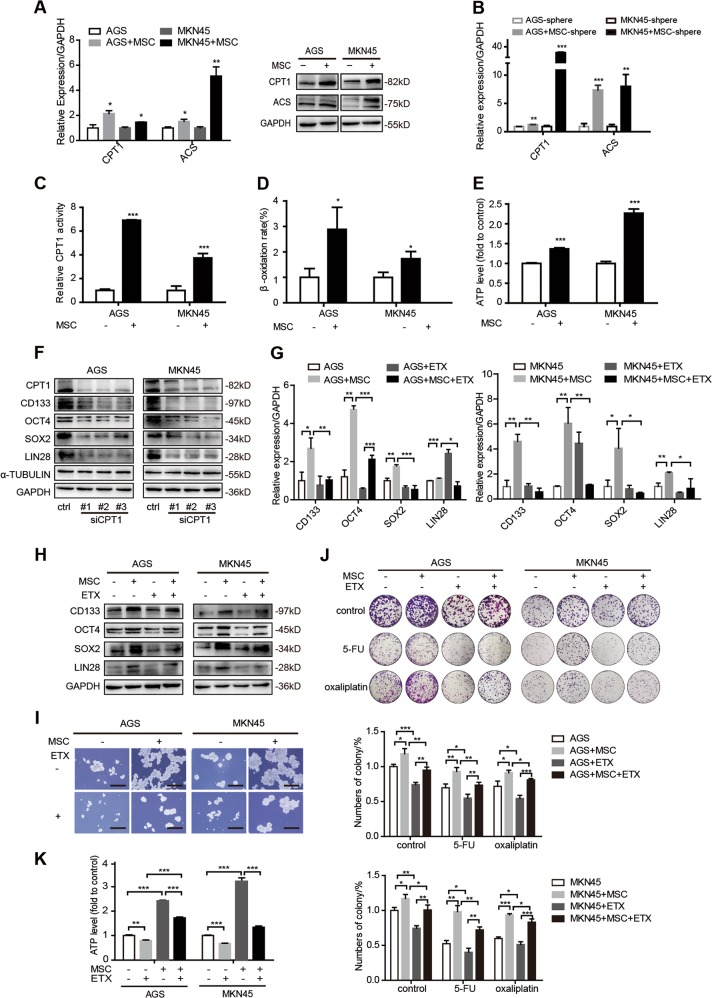
Fatty acid oxidation plays an important role in mesenchymal stem cell (MSC)-induced stemness and chemoresistance. **a** Expression levels of carnitine palmitoyltransferase 1 (CPT1) and acetyl-coenzyme A synthetase (ACS) in AGS and MKN45 co-culture with MSCs compared to culture alone by quantitative real-time polymerase chain reaction (qRT-PCR) and western blotting. **b** qRT-PCR for CPT1 and ACS in AGS and MKN45 sphere culture either alone or with MSCs. **c**–**e** CPT1 enzyme activity (**c**), relative fatty β-oxidation rate (**d**), and ATP levels (**e**) in AGS and MKN45 cells with or without MSCs. **f** Expression levels of stemness-associating genes in AGS and MKN45 transfected with siCPT1. **g**, **h** qRT-PCR (**g**) and western blotting (**h**) for stemness-associating genes in AGS and MKN45 cell culture either alone or with MSCs and with or without 100 μmol/L etomoxir (ETX). **i** Representative images of sphere-formation assay in AGS and MKN45 cell culture either alone or with MSCs and with or without 100 μmol/L ETX for 7 days. Scale bar = 500 μm. **j** Colony-formation assay and the quantitative graph of AGS and MKN45 cell culture either alone or with MSCs and with or without 100 μmol/L ETX when treated with 1 μg/mL 5-florouracil and 3 μg/mL oxaliplatin. **k** ATP level in AGS and MKN45 cell culture either alone or with MSCs and with or without 100 μmol/L ETX for 48 h. *P < 0.05; **P < 0.01; ***P < 0.001

During the past decade, researchers have gained better understanding in the relationship between cancer metabolism and cancer stemness [23–25]. However, the contribution of lipid catabolism to cancer stemness remains unclear. Recent studies have demonstrated that stemness maintenance benefits from FAO by mitochondrial metabolism of FAs [13, 26]. Hence, we wondered whether FAO played an important role in MSC-inducing stemness and chemotherapy resistance. We found that the expression levels of FAO-associated enzymes, carnitine palmitoyltransferase 1 (CPT1) and acetyl-coenzyme A synthetase (ACS), were increased in both GC cells and sphere-forming GC cells when co-cultured with MSCs (Fig. 2a, b). Consistently, FA uptake, CPT1 activity, FAO rate, and ATP level of GC cells were enhanced in the presence of MSCs (Supplementary Fig. 2a and Fig. 2c–e).

To investigate the role of FAO in stemness and chemotherapy resistance, AGS and MKN45 cells were transfected with siCPT1 sequences, which decreased CPT1 mRNA and protein expression (Supplementary Fig. 2b-c). Expression levels of stemness genes were remarkably reduced with silencing of CPT1 (Fig. 2f). MTT (3-(4,5-dimethyl-2-thiazolyl)−2,5-diphenyltetrazolium bromide) assay showed that inhibition of CPT1 expression increased the growth inhibition effect in GC subjected to 5-FU and oxaliplatin, suggesting that silence of CPT1 enhances the sensitivity of GC cells to chemotherapy (Supplementary Fig. 2d).

Next FAO inhibitor etomoxir (ETX), which specifically suppresses CPT1, was employed to further investigate the contribution of FAO to MSC-induced stemness and chemoresistance. We treated GC cells with or without 100 μmol/L ETX for 48 h cultured either alone or with MSCs. As expected, the expression levels of stemness genes and the ability of sphere formation were decreased when GC cells co-cultured with MSCs were exposed to ETX, compared to their control group without ETX (Fig. 2g–i). Colony formation showed that sensitivity to 5-FU and oxaliplatin were increased in GC cells with MSCs when ETX was added (Fig. 2j). Besides, ETX impaired ATP production in GC cells co-cultured with MSCs (Fig. 2k). These results suggest that inhibition of FAO can abrogate the influence of MSCs in promoting stemness and chemoresistance in GC cells, implicating that FAO is required for maintaining MSC-regulated stemness and chemotherapy resistance.

### MACC1-AS1 is induced by MSCs and contributes to stemness and chemoresistance

**Fig. 3:**
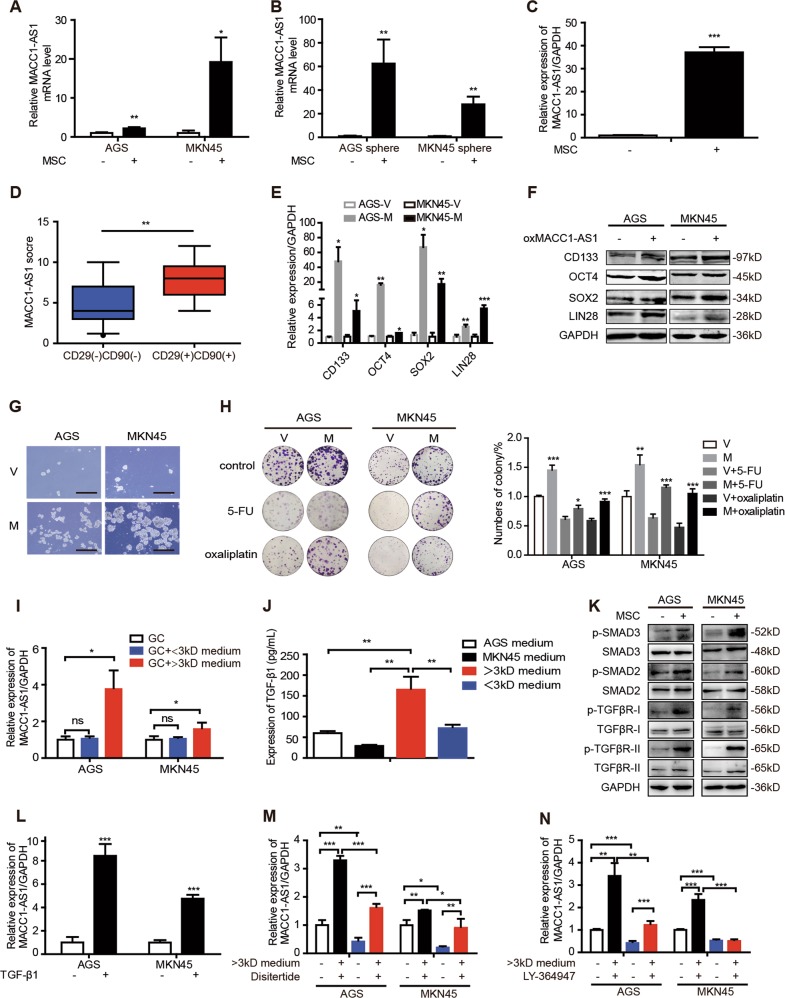
MACC1-AS1 is induced by mesenchymal stem cell (MSC)-derived TGF-β1 and contributes to stemness and chemoresistance. **a**, **b** Expression of MACC1-AS1 in gastric cancer (GC) cells (**a**) and spheres of GC cells (**b**) after co-culture with MSCs. **c** Expression of MACC1-AS1 in the indicated subcutaneous tumor of nude mice, formed by MKN45 cells with or without MSCs. **d** The score of MACC1-AS1 in CD29(−)CD90(−) and CD29(+)CD90(+) GC tissues. **e**, **f** Expression levels of stemness-associating genes was increased in the indicated AGS and MKN45 cells after MACC1-AS1 overexpression by quantitative real-time polymerase chain reaction (**e**) and western blotting (**e**) (V vector, M MACC1-AS1 overexpression). **g** Representative images of sphere-formation assay in AGS and MKN45 cell after overexpressing MACC1-AS1. Scale bar = 500 μm. **h** Colony-formation assay and the quantitative graph of AGS and MKN45 cells treated with 5-florouracil (1 μg/mL) and oxaliplatin (3 μg/mL) after transfected with MACC1-AS1 compared to vector. **i** Expression of MACC1-AS1 in AGS and MKN45 incubated with GC medium, <3 kD MSC-CM or >3 kD MSC-CM. **j** Transforming growth factor (TGF)-β1 concentration in AGS and MKN45 medium, >3 kD MSC-CM and <3 kD MSC-CM measured by enzyme-linked immunosorbent assay. **k** Expression levels of TGF-β receptors and SMAD family in AGS and MKN45 cells after co-culture with MSCs. **l** Expression of MACC1-AS1 in AGS and MKN45 cells treated with TGF-β1 (20 μg/mL) for 24 h. **m**, **n** Expression of MACC1-AS1 in AGS and MKN45 cells incubated with >3 kD MSC-CM treated with TGF-β1 inhibitor disitertide (**m**) and TGFβR-I inhibitor LY-364947 (**n**) for 24 h. *P < 0.05; **P < 0.01; ***P < 0.001

MACC1-AS1 is the antisense RNA of metastasis-associated in colon cancer-1 (MACC1). We previously reported that MACC1-AS1 is overexpressed in GC tissues and facilitates metabolic plasticity through antioxidant production, resulting in a lower ROS load under metabolic stress [18]. Lower ROS levels are critical for maintaining stemness and drug resistance [19–21]. Thus we hypothesized MACC1-AS1 may contribute to stemness and chemoresistance. We found that the expression of MACC1-AS1 was obviously increased both in GC cells and sphere-forming GC cells after co-culture with MSCs (Fig. 3a, b). Additionally, compared to injection of GC cells alone, the expression of MACC1-AS1 in subcutaneous tumor of nude mice was significantly increased in GC cells admixed with MSCs (Fig. 3c). We also performed in situ hybridization (ISH) to evaluate the correlation between MACC1-AS1 expression and co-expression of CD29 and CD90 in GC specimens (Supplementary Fig. 3a). The score of MACC1-AS1 was higher in CD29 and CD90 double-positive GC tissues in comparison to CD29 and CD90 double-negative GC tissues (Fig. 3d). These results indicate that MSCs promote the expression of MACC1-AS1 in GC cells.

Given the promotion of MSCs on MACC1-AS1 expression in GC cells, we next investigated the role of MACC1-AS1 in stemness and chemoresistance. To evaluate whether MACC1-AS1 is functionally involved in GC progression, MACC1-AS1 was overexpressed in stable lentivirus transfected way and the overexpression efficiency was examined (Supplementary Fig. 3b). qRT-PCR and western blotting showed that stemness genes were enhanced after stable transfection with MACC1-AS1 (Fig. 3e, f). The population of CD44^+^ GC cells evaluated by flow cytometric analysis was significantly increased (Supplementary Fig. 3c) and sphere-formation assay showed that sphere-formation capability of GC cells was obviously increased after overexpression of MACC1-AS1 (Fig. 3g). Moreover, overexpression of MACC1-AS1 facilitated the ability of clone formation and reduced growth inhibition when treated with 5-FU and oxaliplatin (Fig. 3h and Supplementary Fig. 3d). To further investigate the role of MACC1-AS1 in chemotherapy, 5FU and oxaliplatin-resistant cell lines were established (Supplementary Fig. 3e). We found that the expression of MACC1-AS1 was gradually increased with augment of 5-FU and oxaliplatin concentration during the establishment of the chemotherapy-resistant cells (Supplementary Fig. 3f). Additionally, the expression levels of stemness genes and FAO relative genes and CPT1 activity were increased in 5-FU and oxaliplatin-resistant cells, compared to 5-FU and oxaliplatin-sensitive cells (Supplementary Fig. 3g–h). Collectively, the results suggest a possible role of MACC1-AS1 in the cross-talk of MSCs and GC cells, participating in MSC-mediated stemness and chemoresistance of GC cells.

### MSC-derived TGF-β1 contributes to MACC1-AS1 upregulation in GC cells

It is reported that MSCs communicate with cancer cells mostly by secreting soluble factors. We collected and fractioned MSC-conditioned medium (CM) based on molecular weight into <3-kD and >3-kD fractions via selective filters and centrifugation. We found that only >3 kD MSC-CM induced MACC1-AS1 upregulation in GC cells (Fig. 3i). These results strongly suggest that the >3-kD fraction of the MSC-CM contains at least one soluble factor that contributes to MSC-mediated stemness and chemoresistance through MACC1-AS1. TGF-β1, a key cytokine that can be secreted by MSCs, has been considered as a critical factor involved in epithelial–mesenchymal transition, metastasis, and stemness [27–29]. Based on the fact that >3 kD MSC-CM induced MACC1-AS1 upregulation, it is likely that TGF-β1 secreted by MSCs contributes to MACC1-AS1 induction.

To verify this possibility, TGF-β1 level from MSC-CM of <3-kD and >3-kD fractions was evaluated by enzyme-linked immunosorbent assays. Compared to MSC-CM of <3 kD and the GC medium collected from AGS and MKN45, the level of TGF-β1 in >3-kD fractions was significantly higher (Fig. 3j). As we have known, TGF-β exerts cellular effects by binding to heterotetrameric complexes of type I and type II serine/threonine kinase receptors (TGFβR-I and TGFβR-II) and activates the downstream SMAD family [30–32]. We found that the expression levels of p-TGFβR-I, p-TGFβR-II, p-SMAD2, and p-SMAD3 were increased when GC cells were co-cultured with MSCs (Fig. 3k), whereas that of SMAD7 were decreased (Supplementary Fig. 3i). These results suggest that TGF-β1 secreted by MSCs contributes to stemness and chemoresistance through binding to TGF-β receptors and activating SMAD2 and SMAD3. To test the effect of TGF-β1 on MACC1-AS1, TGF-β1 (20 μg/mL) was supplemented to GC medium for 24 h. We found that MACC1-AS1 was obviously increased with TGF-β1 treatment (Fig. 3l). Furthermore, TGF-β1 inhibitor disitertide and TGFβR-I inhibitor LY-364947 abrogated the MACC1-AS1 expression in GC cells treated with MSC-CM of >3-kD fraction (Fig. 3m, n). These findings suggest that TGF-β1, as one of the soluble factors secreted by MSCs, is essential for MACC1-AS1 upregulation.

### The role of MACC1-AS1 on stemness and chemoresistance is dependent on FAO

**Fig. 4:**
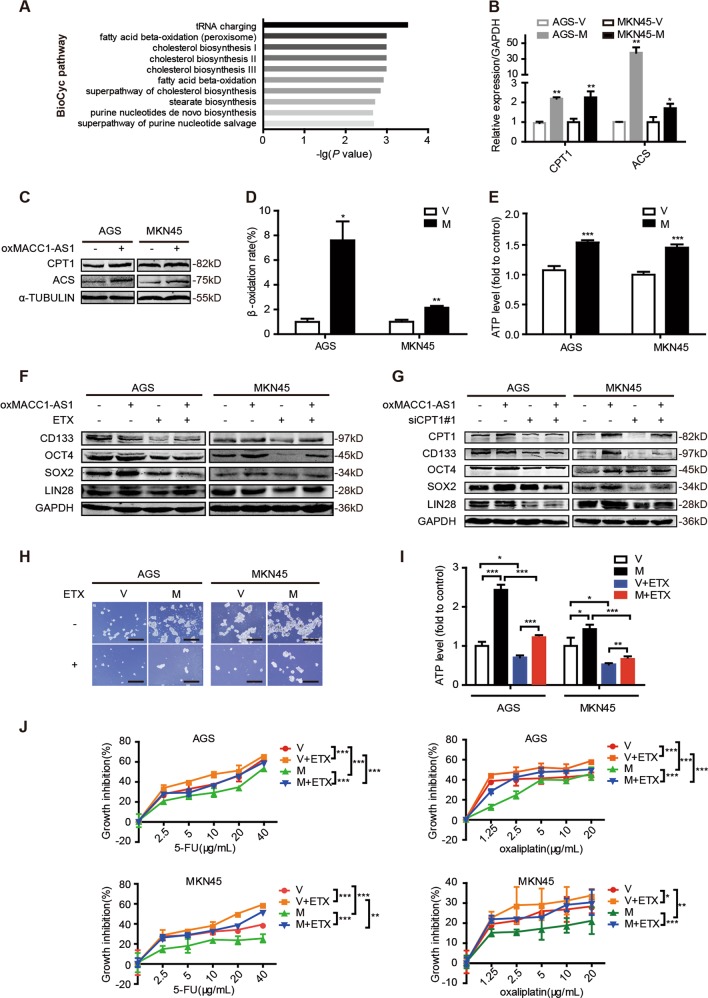
The role of MACC1-AS1 on stemness and chemoresistance is dependent on fatty acid oxidation. **a** The top ten BioCyc metabolic pathway enrichment analysis after MACC1-AS1 overexpression. The horizontal axis represented BioCyc pathway annotation. The vertical axis represented –lg (*P* value). **b**, **c** Quantitative real-time polymerase chain reaction (**b**) and western blotting (**c**) for the expression levels of carnitine palmitoyltransferase 1 (CPT1) and acetyl-coenzyme A synthetase in AGS and MKN45 cells after stable transfection with MACC1-AS1 (M) or vector (V). **d**, **e** Relative fatty β-oxidation rate (**d**) and ATP levels (**e**) in AGS and MKN45 after overexpressing MACC1-AS1. **f** Western blotting for stemness-associating genes in AGS and MKN45 cells after overexpressing MACC1-AS1 and treating with or without 100 μmol/L etomoxir (ETX) for 48 h. **g** Western blotting for expression levels of CPT1 and stemness-associating genes in AGS and MKN45 cells after overexpressing MACC1-AS1 and siCPT1#1 transfection. **h** Representative images of sphere-formation assay in AGS and MKN45 after overexpressing MACC1-AS1 with or without 100 μmol/L ETX for 7 days. Scale bar = 500 μm. **i** Relative ATP levels of AGS and MKN45 cells after overexpressing MACC1-AS1 with or without 100 μmol/L ETX for 48 h. **j** Growth inhibition by MTT (3-(4,5-dimethyl-2-thiazolyl)−2,5-diphenyltetrazolium bromide) assay of AGS and MKN45 cells treated with 5-florouracil and oxaliplatin after overexpressing MACC1-AS1 with or without 100 μmol/L ETX for 48 h. *P < 0.05; **P < 0.01; ***P < 0.001

Based on the existing results, we hypothesized that MACC1-AS1 may be a key participant in FAO-regulated stemness and chemoresistance. We performed RNA sequencing technique (RNA-seq) to profile gene expression after MACC1-AS1 overexpression. BioCyc pathway enrichment revealed that FAO pathway was activated in cancer cells with MACC1-AS1 overexpression (Fig. 4a). Then we found that overexpression of MACC1-AS1 gradually upregulated the levels of CPT1 and ACS as the concentration of MACC1-AS1 transient plasmid increased (Fig. 4b, c and Supplementary Fig. 4a). Consistent results were obtained from detection of FAO rate, ATP production, and FA uptake (Fig. 4d, e and Supplementary Fig. 4b). Next we further assessed whether the promoting effect of MACC1-AS1 in stemness and chemoresistance depended on FAO. As expected, the expression levels of stemness genes were decreased in MACC1-AS1-overexpressing GC cells with 100 μmol/L ETX for 48 h in comparison to MACC1-AS1-overexpressing GC cells without ETX (Fig. 4f and Supplementary Fig. 4c). The same result was obtained by suppression of CPT1 with siRNA#1 (Fig. 4g). Moreover, ETX abrogated the promoting effect of MACC1-AS1 on the ability of self-renewal and reduced mitochondria ATP production accelerated by MACC1-AS1 (Fig. 4h, i). MTT assay showed that ETX treatment partially reversed MACC1-AS1-mediated resistance to 5-FU and oxaliplatin (Fig. 4j). Taken together, FAO is greatly responsible for MACC1-AS1-mediated stemness and chemoresistance of GC cells.

### miR-145-5p is downstream of MACC1-AS1 to promote stemness and chemoresistance through FAO

**Fig. 5:**
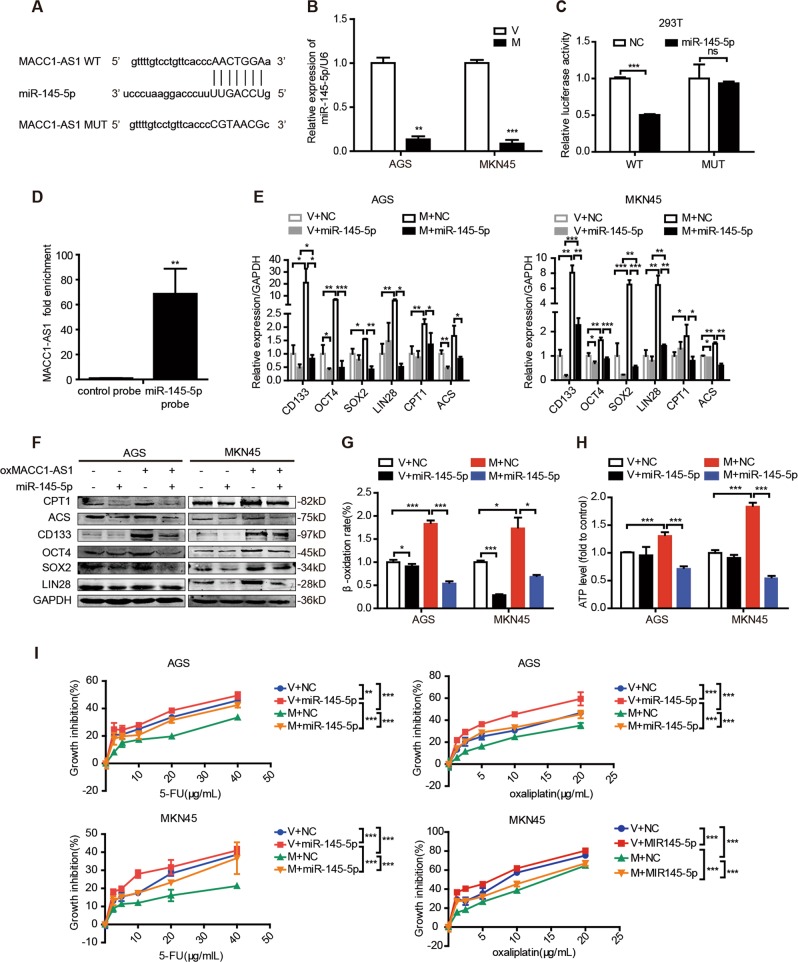
miR-145-5p is downstream of MACC1-AS1 to promote stemness and chemoresistance through fatty acid oxidation (FAO). **a** Schematic diagram of binding sites between MACC1-AS1 and miR-145-5p predicted by LncRNASNP database. **b** Expression of miR-145-5p in AGS and MKN45 cells after stable transfection with MACC1-AS or vector. **c** Luciferase activity in 293T cells when MACC1-AS1 wild-type or MUT vector was co-transfected with miR145-5p mimic or negative control (NC). **d** Quantitative real-time polymerase chain reaction (qRT-PCR) was used to detect the expression of MACC1-AS1 in the miR-145-5p pull-down complex. **e**, **f** qRT-PCR (**e**) and western blotting (**f**) for the expression levels of FAO enzymes and stemness-associating genes in AGS and MKN45 cells after overexpression of MACC1-AS1 with or without transfection with miR-145-5p. **g**, **h** Relative fatty β-oxidation rate (**g**) and ATP levels (**h**) in AGS and MKN45 cells after overexpressing MACC1-AS1 with or without transfection with miR-145-5p. **i** Growth inhibition by MTT (3-(4,5-dimethyl-2-thiazolyl)−2,5-diphenyltetrazolium bromide) assay of AGS and MKN45 cells treated with 5-florouracil and oxaliplatin after overexpressing MACC1-AS1 with or without transfection with miR-145-5p. *P < 0.05; **P < 0.01; ***P < 0.001

Emerging evidences suggest that lncRNAs are likely to function through interaction with microRNAs (miRNAs). To elucidate the mechanism underlying the contribution of MACC1-AS1 to FAO-dependent stemness and chemoresistance, we found that miR-145-5p was one of the target miRNAs of MACC1-AS1 predicted by LncRNASNP database (Fig. 5a). Previous studies have reported that miR-145-5p exerts an inhibitory effect on drug resistance [33, 34] and is correlated with lipid metabolism [35, 36]. Therefore, we wondered whether miR-145-5p played a role in MACC1-AS1-induced stemness and chemoresistance.

First, miR-145-5p expression was significantly decreased after overexpression of MACC1-AS1 and it was decreased as the concentration of MACC1-AS1 transient plasmid increased (Fig. 5b and Supplementary Fig. 5a). Thus dual luciferase reporter assay was employed to further confirm their relationship. The result showed that overexpression of miR-145-5p decreased the reporter activity of MACC1-AS1 wild type (WT) in 293T cell and MKN45 cell but not that of MACC1-AS1 mutant type (MUT), confirming the direct binding sites predicted by LncRNASNP database (Fig. 5c and Supplementary Fig. 5b). For validation, we performed RNA pull down assay to capture RNA complexes associated with miR-145-5p probes. Compared to the control probe, miR-145–5p positive probe successfully pulled down MACC1-AS1 in MKN45 cells (Fig. 5d). Taken together, these data suggest that MACC1-AS1 is able to directly bind to miR-145-5p and regulate its activity.

To determine the role of miR-145-5p in FAO-dependent stemness and chemoresistance, we found that miR-145-5p significantly repressed stemness genes and FAO enzyme expression levels (Supplementary Fig. 5c–d). Moreover, miR-145-5p reduced FAO rate and ATP production and promoted ROS production and sensitivity to 5-FU and oxaliplatin (Supplementary Fig. 5e–h). When MACC1-AS1 and miR-145-5p were co-transfected into GC cells, miR-145–5p remarkably repressed the promoting effect of MACC1-AS1 on the expression levels of stemness genes and FAO enzymes (Fig. 5e, f). In addition, overexpression miR-145-5p significantly reduced the FA uptake, FAO rate, and ATP production induced by MACC1-AS1 overexpression (Supplementary Fig. 5i and Fig. 5g, h). MTT assay showed that miR-145-5p partially rescued the inhibitory impact of MACC1-AS1 on sensitivity of GC cells to 5-FU and oxaliplatin (Fig. 5i). These data suggest that MACC1-AS1/miR-145-5p signaling axis contributes to the FAO-dependent stemness and chemoresistance.

### Inhibition of FAO attenuates MSC-induced chemoresistance in vivo

**Fig. 6:**
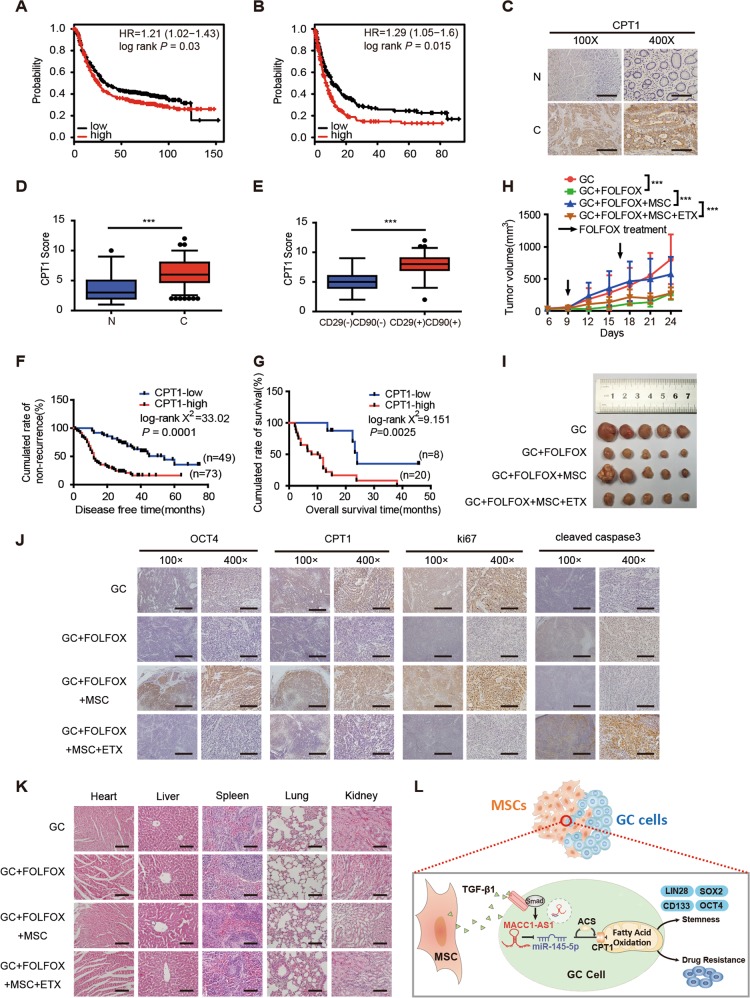
Inhibition of fatty acid oxidation (FAO) attenuates mesenchymal stem cell (MSC)-induced chemoresistance in vivo. **a**, **b** Kaplan–Meier analysis of overall survival (OS) (**a**) and progression-free survival (**b**) corresponding to the expression of carnitine palmitoyltransferase 1 (CPT1) analyzed by the online Kaplan Meier Plotter database (http://kmplot.com/analysis/). **c**, **d** Representative immunohistochemical (IHC) staining of CPT1 (**c**) and CPT1 score (**d**) in adjacent noncancerous and gastric cancer (GC) tissues. Scale bar = 500 μm (magnification: ×100, left panel); Scale bar = 100 μm (magnification: ×400, right panel). **e** CPT1 score in CD29(−)CD90(−) and CD29(+)CD90(+) expression in GC tissues. **f**, **g** Kaplan–Meier analysis of disease-free survival (stage I–III GC patients) and OS (stage IV GC patients) corresponding to the expression of CPT1. **h** Tumor growth of with MKN45 cells (1 × 10^6^ cells) alone (GC) or MKN45 cells treated with FOLFOX regiment weekly (oxaliplatin 6 mg/kg followed 2 h later by 5-florouracil 50 mg/kg and calcium folinatc 90 mg/kg, intraperitoneally (i.p.), GC+FOLFOX) or MKN45 cells in combination with MSCs (5 × 10^6^ cells) treated with FOLFOX regiment (GC+FOLFOX+MSC) or MKN45 cells in combine with MSCs (5 × 10^6^ cells) treated with FOLFOX regiment plus ETX (40 mg/kg, i.p., every other day, GC+FOLFOX+MSC+ETX). Tumor volumes were calculated every 3 days (*n* = 5). **i** Representative images of transplanted subcutaneous tumors. **j**, **k** Representative images of OCT4, CPT1, ki67, and cleaved caspase 3 in subcutaneous tumors of xenograft nude mice by IHC staining (**j**) and hematoxylin–eosin staining of the hearts, livers, spleens, lungs, and kidneys in subcutaneous tumors (**k**). Scale bar = 500 μm (magnification: ×100, left panel); scale bar = 100 μm (magnification: ×400, right panel). **l** Schematic representation of the pathway that MSCs secreted TGF-β1 and induced lncRNA MACC1-AS1 expression in GC cells, which promoted FAO-dependent stemness and chemoresistance through miR-145-5p. *P < 0.05; **P < 0.01; ***P < 0.001

We found that higher CPT1 expression in GC patients was associated with shorter OS and progression-free survival (PFS) analyzed by the online Kaplan Meier Plotter database (Fig. 6a, b). Then we analyzed the expression of CPT1 in primary GC samples and matched adjacent nontumorous gastric tissues by IHC. Compared to the matched adjacent nontumorous gastric tissues, CPT1 score was higher in GC (Fig. 6c, d). It was also higher in CD29 and CD90 double-positive GC tissues in comparison to CD29 and CD90 double-negative GC tissues (Fig. 6e). Moreover, higher CPT1 expression was linked to worse DFS in stage I–III patients and worse OS in stage IV patients (Fig. 6f, g).

We further investigated whether inhibition of FAO attenuated MSC-induced chemoresistance in vivo. FOLFOX regiment, composed of 5-FU, oxaliplatin, and calcium folinate, is the first-line therapy of metastatic GC [4]. The results showed that the tumor volumes of FOLFOX-treated mice were remarkably decreased compared to mice without FOLFOX treatment. MSCs remarkably abolished the antitumor effects of FOLFOX, but intraperitoneal (i.p.) injection with ETX significantly reduced the tumor volumes (Fig. 6h, i). Furthermore, IHC staining of tumors showed that the expression levels of OCT4, CPT1, and proliferation-related nuclear antigen ki-67 were decreased after ETX treatment, while marker of apoptosis cleaved caspase 3 was increased (Fig. 6j). No significant difference was observed in tissues and structures between groups treated with or without ETX by hematoxylin–eosin staining of the lungs, livers, spleens, kidneys, and hearts of xenograft nude mice (Fig. 6k). Taken together, these results suggest that inhibition of FAO attenuates MSC-induced chemoresistance in vivo.

## Discussion

Stroma cells in tumor microenvironment were recognized as important contributors in facilitating the development of chemotherapy resistance. As an important component of tumor environment, MSCs can promote chemotherapy resistance by secreting protective cytokines or even generating genetic mutations and altering transcriptional expression to help cancer cells overcome the anticancer effect of chemotherapeutic agents [8, 37]. Recently, the role of tumor metabolism has been increasingly recognized in the field of chemoresistance in tumors. It was reported that MSCs can secrete polyunsaturated FAs KHT and 16:4(n­3) and subsequently induce resistance to platinum-­based chemotherapy [7]. Thus MSCs play a significant role in metabolic reprogramming in regulation of chemotherapy resistance.

Our findings demonstrated that MSCs promoted GC cell self-renewal and chemoresistance through FAO. FAO inhibitor ETX remarkably reversed the stemness features and resistance to 5-FU and oxaliplatin, which indicated that GC cells undergo metabolic reprogramming when interacting with MSCs. Actually, there is little known about the role of MSCs on FAO in cancer cells. To our knowledge, FAs are important energy sources for cancer cells, as FAO in mitochondria provides 2.5 times as much ATP in comparison with oxidation of glucose [38]. We presented evidence that MSCs promoted FAO and mitochondrial ATP production to maintain stemness and chemoresistance in GC cells, which suggests that MSCs promote ATP production in GC cells to satisfy the demand of overcoming the chemotherapeutic agents. Our study also showed an elevated CPT1 expression in GC tissues and that was linked to worse DFS and OS in GC patients. Inhibition of FAO by ETX prominently reversed the MSC-induced FOLFOX resistance in vivo, which implicates that chemotherapeutic drugs in combination with agents targeting cellular FAO can be a feasible approach for overcoming chemoresistance in GC.

Researches on the metabolic phenotype of CSCs and drug-resistant cells have been getting increasing attention in recent years. In our study, we found that silencing CPT1, FAO rate-limiting enzyme, decreased stemness gene expression levels and resistance to 5-FU and oxaliplatin. Metabolic reprogramming of cancer cells toward FAO benefits stemness maintenance [13, 26] and this alteration is able to modulate chemoresistance by maximizing ATP production [39]. On the other hand, FAO is an important source of NADPH [10]. Inhibition of FAO decreased NADPH and elevated intracellular ROS and eventually induced cancer cell death [12]. Recent studies have showed that CSCs tend to have low ROS level, which is crucial to maintain their self-renewal and stemness properties [19, 40, 41]. Thus upregulating antioxidant capacity to maintain low ROS may contribute to stemness and chemoresistance in GC cells.

Increasing evidence has indicated that lncRNAs are involved in cancer stemness and drug resistance [15–17]. We previously reported that MACC1-AS1 is overexpressed in GC tissues and facilitates metabolic plasticity through increased glutathione and NADPH levels and a lower ROS load under metabolic stress [18], which are critical for maintaining stemness and drug resistance [19–21]. Here we showed that MSCs promoted MACC1-AS1 expression in GC cells and MACC1-AS1 positively regulated FAO-dependent stemness and chemoresistance, suggesting the important role of MACC1-AS1 in the cross-talk between MSCs and GC cells. Moreover, we found TGF-β1 was the upstream molecular of MACC1-AS1 secreted by MSCs. Previous studies have demonstrated that, when interacting with cancer cells, MSCs release elevated levels of TGF-β, which supports the growth and invasion abilities of cancer cells [42, 43]. Meanwhile, the role of TGF-β in maintaining stem cell self-renewal is well established [27–29]. In our study, MSCs secreted TGF-β1 and led to subsequent upregulation of MACC1-AS1 in GC cells through activation of SMAD2 and SMAD3. TGF-β1 inhibitor disitertide and TGFβR-I inhibitor LY-364947 abrogated the MACC1-AS1 expression in GC cells, suggesting that targeting TGF-β signaling pathway may be a potential strategy to inhibit MSC-induced stemness and chemoresistance.

Additionally, we found that MACC1-AS1 promoted stemness and chemoresistance through antagonizing miR-145-5p. The localization of lncRNAs is important for their function and MACC1-AS1 mainly locates in the cytoplasm [18], where lncRNAs typically participate in posttranscriptional regulation by interacting with microRNAs [44, 45]. Luciferase reporter assay and pull down assay demonstrated that MACC1-AS1 directly bound to miR-145-5p and MACC1-AS1 negatively regulated miR-145-5p. However, the binding sites between miR-145-5p and CPT1 cannot be found. Interestingly, our results showed that miR-145-5p depressed CPT1 expression. When MACC1-AS1 and miR-145-5p were co-transfected into GC cells, miR-145-5p partially reversed the promoting effect of MACC1-AS1 on expression of CPT1, which strongly suggested that they are connected through some regulatory networks. Besides, previous studies have reported that miR-145-5p exerts an inhibitory effect on drug resistance. miR-145-5p can directly target multidrug resistance protein 1 to increase the toxicity of chemotherapeutic drug [46]. Moreover, miR-145-5p was able to target stemness genes to decrease chemoresistance. miR-145 bound to the SOX2 mRNA 3′-untranslated region (3′UTR) and affected the anticancer response of isorhapontigenin in glioblastoma [47]. In our study, we found that miR-145-5p increased ROS production and cell apoptosis in GC cells, which indicated that ROS-induced apoptosis was one of the important mechanisms in MACC1-AS1-promoted chemoresistance.

In summary, our study demonstrated that MSCs secreted TGF-β1 and induced lncRNA MACC1-AS1 expression in GC cells, which promoted FAO-dependent stemness and chemoresistance through miR-145-5p. Combining chemotherapeutic drugs with targeting cellular FAO may be a promising strategy to overcome chemotherapeutic resistance (Fig. 6l).

## Materials and methods

### Reagents

CPT1 (#15184–1-AP), CD29 (#12594–1-AP), CD133 (#18470–1-AP), OCT4 (#11263–1-AP), SOX2 (66411–1-Ig), LIN28 (#60344–1-Ig), TGF-β receptor I (55391–1-ap), SMAD2 (#12570–1-ap), SMAD3 (#25494–1-ap), GAPDH (#10494–1-AP), and α-Tubulin antibodies (#11224–1-AP) were obtained from Proteintech (Wuhan, China). ACS (#ab133664), TGF-β receptor II (ab184948), phosphor-TGF-β receptor II (Ser225, #ab183037), and CD90 (#ab133350) antibodies were purchased from Abcam (Cambridge, MA, USA). Phosphor-SMAD2 (Ser465/467, #138D4) and phosphor-SMAD3 (Ser423/425, #C25A9) antibodies were obtained from Cell Signaling Technology (Boston, MA, USA). Phosphor TGF-β receptor I (Ser-165, #orb335748) antibody was from Biorbyt (Shanghai, China). TGF-β1 (#AF-100–21C-10), epidermal growth factor (EGF; #AF-100–15–100), fibroblast growth factor (FGF; #100–18B-10) were from PeproTech (Rocky Hill, NJ, USA), while B27 (#17504044) was from Gibco (Grand Island, NY, USA). ETX and TGF-β1 inhibitors Disitertide (#HY-P0118) and TGFβR-I inhibitor LY-364947 (#HY-13462) were provided by MedChemExpress (NJ, USA). Lipofectamine 2000 reagent was obtained from Invitrogen (#11668019, CA, USA). 5-FU, oxaliplatin, and calcium folinatc was from Xudong (Shanghai, China), Sanofi (Shanghai, China), and Yaoyou (Chongqing, China), respectively. Fluorescein isothiocyanate (FITC) Mouse Anti-Human CD44 was provided by BD Biosciences (#560977, NY, USA).

### Cell lines and cell culture

The GC cell lines AGS was purchased from KeyGene Biotechnology Development Company (Nanjing, China). MKN45 was obtained from Cellcook Biotechnology Company (Guangzhou, China). AGS and MKN45 cancer cells stably overexpressing MACC1-AS1 were constructed with lentivirus combined with pLenti-EF1a-LUC-F2A-CMV-MCS vector or blank vector and selected with 2 μg/mL puromycin at 72 h after infection. The cells mentioned above were cultured with RPMI 1640 medium with 10% fetal bovine serum (Thermo scientific Hyclone, USA) under conditions of 5% CO_2_ at 37 °C. Adult bone marrow MSCs, purchased from Cyagen Biosciences (Guangzhou, China), were cultured in adult bone marrow MSC medium at 37 °C under 5% CO_2_. Authenticity of the cells was regularly tested by short tandem repeat. MSCs and GC cells were grown in a Corning chamber where MSCs were cultured in the top insert at a density of 2 × 10^5^ cells and GC cells were cultured in the bottom well at a density of 1.5 × 10^5^ cells for 48 h. The two populations were separated by a 0.4-µm porous membrane that allows for soluble factor exchange.

### Generation of 5-FU- and oxaliplatin-resistant cell lines

The 5-FU- and oxaliplatin-resistant cell lines were established by continuously exposing GC cells to increasing concentrations of 5-FU (0.125–8 μg/mL for AGS and 0.5–12 μg/mL for MKN45) and oxaliplatin (1–12.5 μg/mL for AGS and 0.8–8 μg/mL for MKN45).

### CPT1 activity assay

Cell mitochondrial proteins were extracted using the Cell Mitochondria Isolation Kit (Beyotime, Shanghai, China) and quantified using BCA assay (Cpr). CPT1 activity was measured using a CPT1 Spectrophotometric Detection Kit (Zikerbio, Guangzhou, China). Briefly, CPT1 activity was determined spectrophotometrically by the reaction between DNTB and CoA-SH released from palmitoyl CoA. Mitochondrial proteins (40 μL) were mixed with 880 μL carnitine (reagent 5) and 40 μL palmitoyl CoA (reagent 6) in a clean cuvette and the absorbance of the mixtures was measured at 412 nm by spectrophotometry (OD1). The reaction mixtures were incubated at 37 °C for 2 min followed by the detection of absorbance at 412 nm again (OD2). The difference between the absorbances was used to measure the release of CoA-SH. CPT1 activity was calculated according to the following formula: nmol/min/mg = 880 × (OD2 − OD1)/Cpr.

### Clinical samples

A total of 151 formalin-fixed and paraffin-embedded GC tissue samples were obtained from patients who were diagnosed with GC in Nanfang Hospital (Guangzhou, China) between January 2006 and December 2010. Patients’ staging data were defined according to the Seventh Edition of the AJCC Cancer Staging Manual: Stomach (2010) [48]. The use of human tissue samples and clinical data was approved by the ethics committee of Nanfang Hospital Ethics Review Board (Guangzhou, China). Informed consent was obtained from all patients.

### ISH staining and IHC staining

ISH was performed as previously described [18]. Briefly, MACC1-AS1 was detected using a double digoxigenin-labeled mercury locked nucleic acid probe (5′DigN/TCAATGCAGATCTAATACTCCT/3′Dig_N) (miRCURY LNA™, Exiqon, Vedbaek, Denmark). IHC staining was applied to detect the expression levels of CD29, CD90, OCT4, CPT1, cleaved caspase 3, and ki67. The staining intensity was scored as 0 (negative), 1 (weak), 2 (medium), or 3 (strong), whereas the staining extent was scored as 0 (0% of the staining area), 1 (1–25%), 2 (26–50%), 3 (51–75%), and 4 (76–100%). The final scores of the targeted protein expression were calculated by the products of the staining intensity and extent scores, low expression group (score 0–5), and high expression group (score 6–12).

### Animal experiments

Female BALB/C nude mice (4-week old) were purchased from the Experimental Animal Center of Nanfang Hospital (Guangzhou, China) and were assigned randomly into different groups. The study was approved by Institutional Animal Ethics Committee of Nanfang Hospital. For in vivo limiting dilution assay, serial dilutions of MKN45 cell suspensions (5 × 10^5^, 5 × 10^4^, and 5 × 10^3^ cells) admixed with or without 5 × 10^6^ MSCs were injected subcutaneously into nude mice. After 6 weeks, the mice were euthanized and tumor formation was examined. Tumor-initiating cell frequencies were determined using the ELDA webtool (http://bioinf.wehi.edu.au/software/elda). To assess the role of FAO in MSC-induced chemoresistance, 1 × 10^6^ MKN45 cells were injected subcutaneously into the right flank of nude mice. Tumor volume measurements were evaluated by digital calipers and calculated by the formula (π)/6 × (large diameter) × (small diameter) [2]. When tumor volume reached to 50–100 mm^3^, FOLFOX regiment was administered i.p. weekly (oxaliplatin 6 mg/kg followed 2 h later by 5-FU 50 mg/kg and calcium folinatc 90 mg/kg). ETX (40 mg/kg) was administered i.p. every other day (*n* = 5). Mice were weighed and tumor volumes were scaled every 3 days by one of the authors who did not know any of the experiment groups throughout the duration of the study.

### Bioinformatics analysis

Gene expression data and clinical data of 384 GC patient samples and 37 normal gastric samples were downloaded from TCGA (https://cancergenome.nih.gov/) on November 8, 2017. The expression values of CD29 (ITGB1) and CD90 (THY1) were separated into two parts according to the median. The RFS of patients sharing expression values greater than the median of CD29 and CD90 were compared with those patients sharing expression values below the median of these genes using the log-rank test. RNA-seq was applied to profile gene expression after MACC1-AS1 overexpression. R package limma was employed to perform gene expression analysis. Fold change > 2 and adjusted *P* value <0.05 were set as the threshold for significant differential expression. KOBAS software was used to perform pathway enrichment analysis for differentially expressed genes to explore their biological functions using BioCyc metabolic database. Kaplan–Meier analysis of OS and PFS corresponding to the expression of CPT1 was analyzed by the online Kaplan Meier Plotter database (http://kmplot.com/analysis/).

### MTT assay

Cells were plated in 96-well plates and exposed to various concentrations of oxaliplatin or 5-FU for 48 h. Thiazolyl blue (MTT) was added to the cells and incubated for 4–6 h, followed by replacement of 150 μL per well dimethyl sulfoxide as previously described [49]. Absorbance was measured at 570 nm using a SpectraMax M5 microplate reader (Molecular Devices, Sunnyvale, CA).

### Quantitative real-time polymerase chain reaction (qRT-PCR)

Total RNA was extracted from specific GC cells using the Trizol Kit according to the manufacturer’s instructions, followed by reverse transcription with Takara RT reagent (Takara Bio, Shiga, Japan). qRT-PCR was performed using a LightCycler 480 system Version 1.5 (Roche, Penzberg, Germany) as described in our previous study [49]. The sequences of primers used for qRT-PCR are listed in Table 2.

**Table 2:** Primer sequences involved in qRT-PCR

Gene	Sequences (5′−3′)
GAPDH-F	ACCCAGAAGACTGTGGATGG
GAPDH-R	TCTAGACGGCAGGTCAGGTC
MACC1-AS1-F	GCCAGTCAGAAAATGAGGAAC
MACC1-AS1-R	CCAGTTGGGTGAACAGGAC
OCT4-F	CTTGAATCCCGAATGGAAAGGG
OCT4-R	GTGTATATCCCAGGGTGATCCTC
CD133-F	AGTCGGAAACTGGCAGATAGC
CD133-R	GGTAGTGTTGTACTGGGCCAAT
SOX2-F	GCCGAGTGGAAACTTTTGTCG
SOX2-R	GGCAGCGTGTACTTATCCTTCT
LIN28-F	TGCGGGCATCTGTAAGTGG
LIN28-R	GGAACCCTTCCATGTGCAG
CPT1A-F	TCCAGTTGGCTTATCGTGGTG
CPT1A-R	TCCAGAGTCCGATTGATTTTTGC
ACS-F	GATGACTTGTACCATTGGTCCG
ACS-R	ACGTGAGAAGACAATTCCACTG

*qRT-PCR* quantitative real-time polymerase chain reaction

### Western blotting

Western blotting was performed as previously described [18] using anti-ACS, anti-CPT1, anti-CD133, anti-OCT4, anti-SOX2, and anti-LIN28 antibodies. GAPDH and α-Tubulin served as the loading controls. Immunoblots were detected with fluorophore-conjugated goat anti-rabbit or anti-mouse secondary antibodies (LI-COR, Lincoln, NE, USA) by an Odyssey imaging system (LI-COR).

### Small interfering RNA (siRNA) transfection

The GC cell lines AGS and MKN45 were transfected with targeted or control siRNA using Lipofectamine 2000 as previously reported [49]. The sequences of CPT1 siRNA were GAGAGAACCTCATCAATTT, GGAGGAAATCAAACCAATT, and GCCTTTACGTGGTGTCTAA. The efficiency of sequences were verified by qRT-PCR and western blotting.

### Flow cytometric analysis

GC cells were harvested and washed twice with phosphate-buffered saline (PBS). The cells were fixed with bovine serum albumin and stained with FITC Mouse Anti-Human CD44. Flow cytometry was analyzed with a FACS Aria II instrument (BD Biosciences).

### Sphere-formation assay

Cells were seeded into 6-well low attachment plates (Corning, Shanghai, China) as single-cell suspension at 1 × 10^3^ cells per well and cultured with Dulbecco’s modified Eagle’s medium-F12 medium Gibco (NY, USA) containing 2% B-27, 20 ng/mL EGF, and 10 ng/mL FGF. Seven days later, spheroids were observed and photographed with inverted microscope (Olympus, Japan). All assays were performed in three replicates.

### Colony formation

Cells were seeded into 6-well plates (BIOFIL, Guangzhou, China) at a concentration of 5 × 10^2^ cells per well and cultured for 14 days. Culture medium was replaced every 4 days. The colonies were fixed with 4% paraformaldehyde for 30 min, stained with 0.1% crystal violet for 30 min, and then photographed.

### Detection of FA β oxidation rate

Cells were collected and mitochondria were extracted using the Cell Mitochondria Isolation Kit (Beyotime, China). FA β oxidation rate was measured using a FA β Oxidation Rate Colorimetric Detection Kit (Haling, Shanghai, China). Briefly, a clean cuvette was filled with 750 μL reagent A, 100 μL reagent B, 50 μL reagent C, and 50 μL reagent D and the mixture was incubated at 25 °C for 3 min after turning it upside down. Fifty μL mitochondria solution or 50 μL negative reagent was added to the mixture for sample detection or blank control. Immediately, absorbance was measured at 420 and 470 nm for 5 min by dual-wavelength spectrophotometry. The β oxidation rate was calculated according to the following formula: μmol/min/mg = [(sample reading − blank reading) × 1 mL] ÷ (0.5 mg × 105 × 5 min); sample/blank reading = (OD420 − OD470)_0 min_ − (OD420 − OD470)_5 min_. All assays were performed in three replicates.

### Detection of cellular ATP levels

Cells were collected and schizolysized with 200 μL lysis buffer, followed by centrifugation at 1.2 × 10^4^ × *g* for 5 min. The supernatant was transferred for ATP detection using a Firefly Luciferase-based ATP Assay Kit (Beyotime, China). Twenty μL of each supernatant was added to 10 μL ATP detection working dilution. Luminance (relative luminsescence unit) was measured using a SpectraMax M5 microplate reader (Molecular Devices, Sunnyvale, CA). The protein levels of each sample was also detected using BCA assay and standard curves of ATP and protein were generated. Cellular relative ATP levels were calculated by the ratio of ATP value to protein value.

### Dual-luciferase reporter assay

The MACC1-AS1–3′UTR vectors, including pMIR-REPORT-MACC1-AS1–3′UTR WT and pMIR-REPORT-MACC1-AS1–3′UTR mutant type (MUT), were purchased from OBiO (Shanghai, China). Cells were seeded with a concentration of 5000 per well in 96-well plates and transfected with MACC1-AS1–3′UTR vectors, renilla luciferase plasmid, miR-145-5p mimics, or miR-NC using Lipofectamine 2000. The cells were harvested after 36 h and the luciferase activity was assessed using a dual-luciferase reporter assay system (Promega, Madison, USA) according to the manufacturer’s instructions.

### RNA pull down assay

The biotinylated miR-145-5p probes were synthesized by CLOUD-SEQ Company (Shanghai, China). The miR-145-5p probe: 5′ GUCCAGUUUUCCCAGGAAUCCCU-Biotin 3′; and the control probe: 5′ AGGGATTCCTGGGAAAACTGGAC-Biotin 3′. The cells were washed with PBS twice and incubated in lysis buffer on ice for 10 min. The lysates were precleared by centrifugation. The probes were incubated with streptavidin-coated magnetic beads to generate probe-coated magnetic beads. The lysates were incubated with probe-coated beads and washed with the wash buffer. The RNA complexes bound to the beads were eluted and subjected to qRT-PCR for detection of the expression of relative MACC1-AS1.

### Statistical analysis

All the experimental data were expressed as means ± SD and statistically analyzed with the SPSS v. 20.0 software (SPSS Inc., Chicago, IL, USA). For comparisons, two-tailed Student’s *t* test, Fisher’s exact test, one-way analysis of variance test, and Dunnett’s test were performed. Survival and recurrence rates were analyzed with Kaplan–Meier’s method and examined by log-rank test. *P* values < 0.05 were considered statistically significant.

## Supplementary information

Supplemental information

Supplemental Figure 1

Supplemental Figure 2

Supplemental Figure 3

Supplemental Figure 4

Supplemental Figure 5
