# Supplementary material for: MSC-regulated lncRNA MACC1-AS1 promotes stemness and chemoresistance through fatty acid oxidation in gastric cancer
Source: Oncogene. 2019 Feb 11;38(23):4637–54. doi: 10.1038/s41388-019-0747-0 (PMC6756048; doi:10.1038/s41388-019-0747-0)
Supplement: Supplementary file 1 — Supplemental information [file 41388_2019_747_MOESM1_ESM.docx]

Supplementary Figure 1

(A-B) qRT-PCR (A) and western blotting (B) for stemness-associating genes in AGS and MKN45 with or without co-culture with MSCs. (C) Flow cytometric analysis for the population of CD44 positive (CD44^+^) AGS and MKN45 with or without co-culture with MSCs. (D) Representative IHC staining of OCT4 in adjacent noncancerous and gastric cancer tissues. Scale bar = 500μm (magnification: 100×, left panel); Scale bar = 100μm (magnification: 400×, right panel). (E) The score of OCT4 was in CD29(-)CD90(-) and CD29(+)CD90(+) expression in GC tissues. **P* < 0.05; ** *P* < 0.01; *** *P* < 0.001.

Supplementary Figure 2

(A) Fatty acid uptake of AGS and MKN45 cells after co-culture with MSCs. (B-C) CPT1 expression of AGS and MKN45 cells transfected with three siRNA sequences (siCPT1#1, siCPT1#2, siCPT1#3) by qRT-PCR (B) and western blotting (C). (D) Growth inhibition by MTT assay of AGS and MKN45 treated with 5-FU and oxaliplatin after transfection with siCPT1#1 and siCPT1#2. **P* < 0.05; ** *P* < 0.01; *** *P* < 0.001.

Supplementary Figure 3

(A) Representative images of ISH staining of MACC1-AS1 in adjacent noncancerous and gastric cancer tissues. Scale bar = 500μm (magnification: 100×, left panel); Scale bar=100μm (magnification: 400×, right panel). (B) The efficiency of stable transfection in AGS and MKN45 cell. (C) Flow cytometric analysis for the population of CD44^+^ AGS and MKN45 cells treated with MACC1-AS1 or vector. (D) Growth inhibition by MTT assay of AGS and MKN45 cells treated with 5-FU and oxaliplatin after overexpression of MACC1-AS1. (E) MTT assay for growth inhibition of GC cells and 5-FU and oxaliplatin-resistant GC cells treated with 5-FU and oxaliplatin. (AGS-5FUR, 5-FU-resistant AGS; AGS-OXR, oxaliplatin-resistant AGS; MKN45-5FUR, 5-FU-resistant MKN45; MKN45-OXR, oxaliplatin-resistant MKN45). (F) Relative MACC1-AS1 expression in AGS and MKN45 when exposed to different of concentration 5-FU and oxaliplatin during the establishment of 5-FU and oxaliplatin-resistant resistant GC cells. (G-H) Expressions of stemness genes and FAO relative enzymes (G) and CPT1 activity (H) in GC cells and chemotherapeutic drugs-resistant GC cells. (I) SMAD7 expression in AGS and MKN45 when co-culture with MSCs. **P* < 0.05; ** *P* < 0.01; *** *P* < 0.001.

Supplementary Figure 4

(A) qRT-PCR for CPT1 and ACS expressions in AGS and MKN45 cells after transfection of different concentration of MACC1-AS1 transient plasmid. (B) Fatty acid uptake in AGS and MKN45 cells after overexpressing MACC1-AS1 (M, MACC1-AS1; V, vector). (C) qRT-PCR for stemness-associating genes in AGS and MKN45 cells after overexpressing MACC1-AS1 and treating with or without 100μmol/L ETX for 48h. **P* < 0.05; ** *P* < 0.01; *** *P* < 0.001.

Supplementary Figure 5

(A) Expression of miR-145-5p in AGS and MKN45 cells after transfection of different concentration of MACC1-AS1 transient plasmid. (B) Luciferase activity in MKN45 cells when MACC1-AS1 WT or MUT vector was co-transfected with miR145-5p mimic or negative control (NC). (C-D) qRT-PCR (C) and western blotting (D) for expressions of FAO enzymes and stemness-associating genes in AGS and MKN45 cells transfected with miR-145-5p mimic or NC. (E-F) Relative fatty β-oxidation rate (E) and ATP levels (F) in AGS and MKN45 transfected with miR-145-5p mimic or NC. (G) Growth inhibition by MTT assay of AGS and MKN45 cells treated with 5-FU and oxaliplatin after transfected with miR-145-5p mimic or NC. (H) ROS production of AGS and MKN45 cells treated with 5-FU and oxaliplatin after transfected with miR-145-5p mimic or NC. (I) Fatty acid uptake in AGS and MKN45 cells after overexpression of MACC1-AS1 with or without transfection with miR-145-5p. **P* < 0.05; ** *P* < 0.01; *** *P* < 0.001.
