# Supplementary figures and images for: MSC-regulated lncRNA MACC1-AS1 promotes stemness and chemoresistance through fatty acid oxidation in gastric cancer
Source: Oncogene. 2019 Feb 11;38(23):4637–54. doi: 10.1038/s41388-019-0747-0 (PMC6756048; doi:10.1038/s41388-019-0747-0)

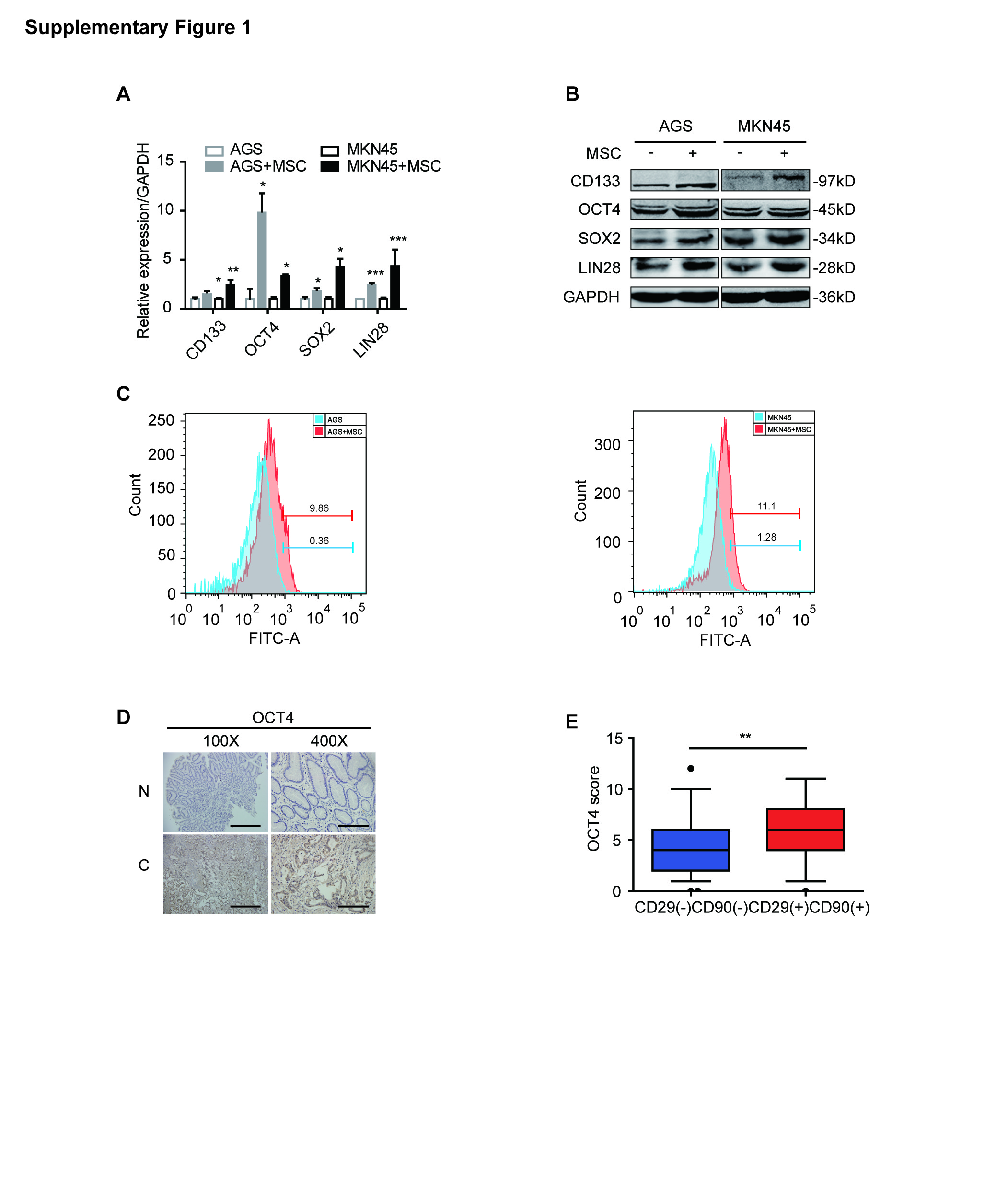

Supplement: Supplementary file 2 — Supplemental Figure 1 [file 41388_2019_747_MOESM2_ESM.jpg]

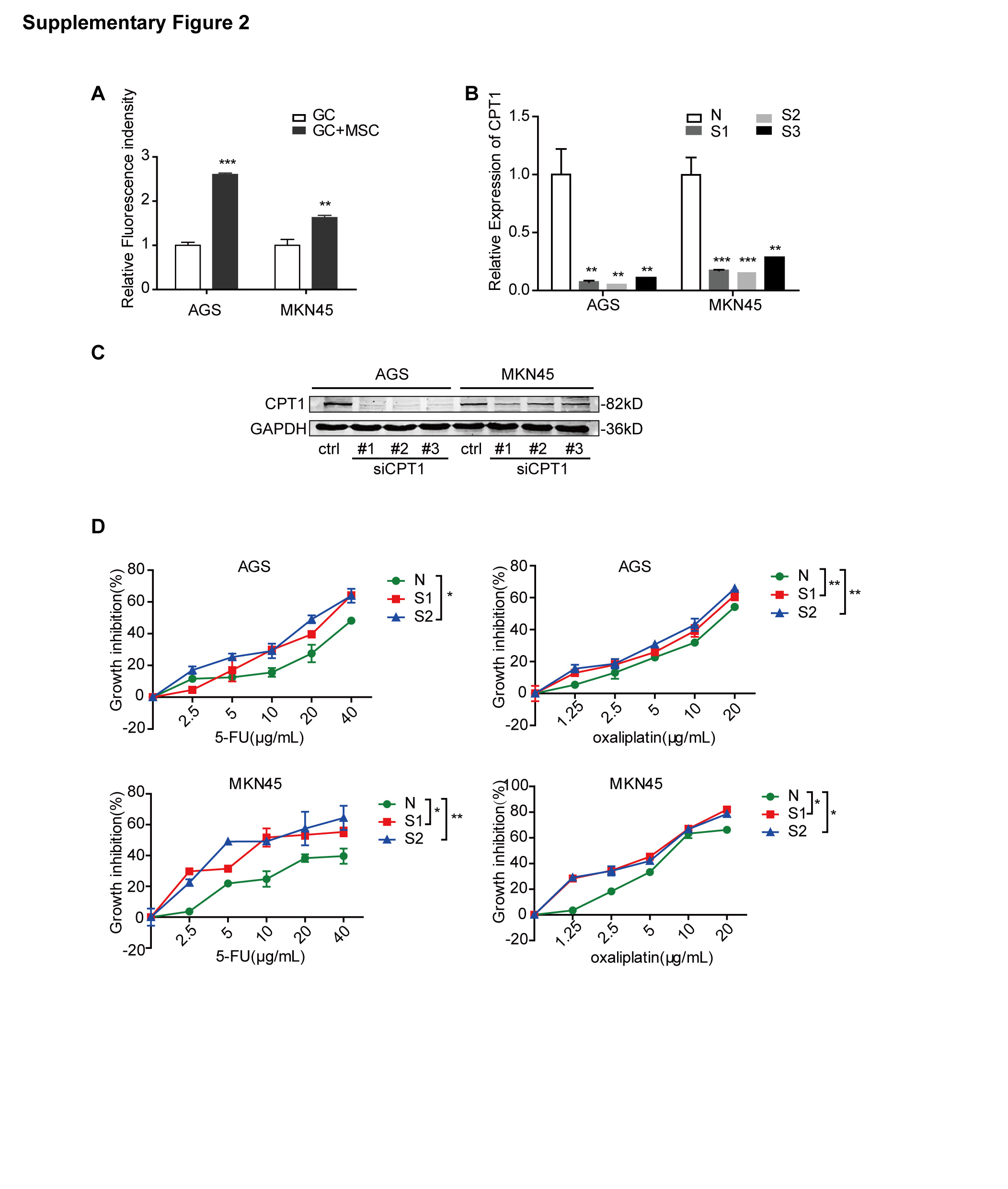

Supplement: Supplementary file 3 — Supplemental Figure 2 [file 41388_2019_747_MOESM3_ESM.jpg]

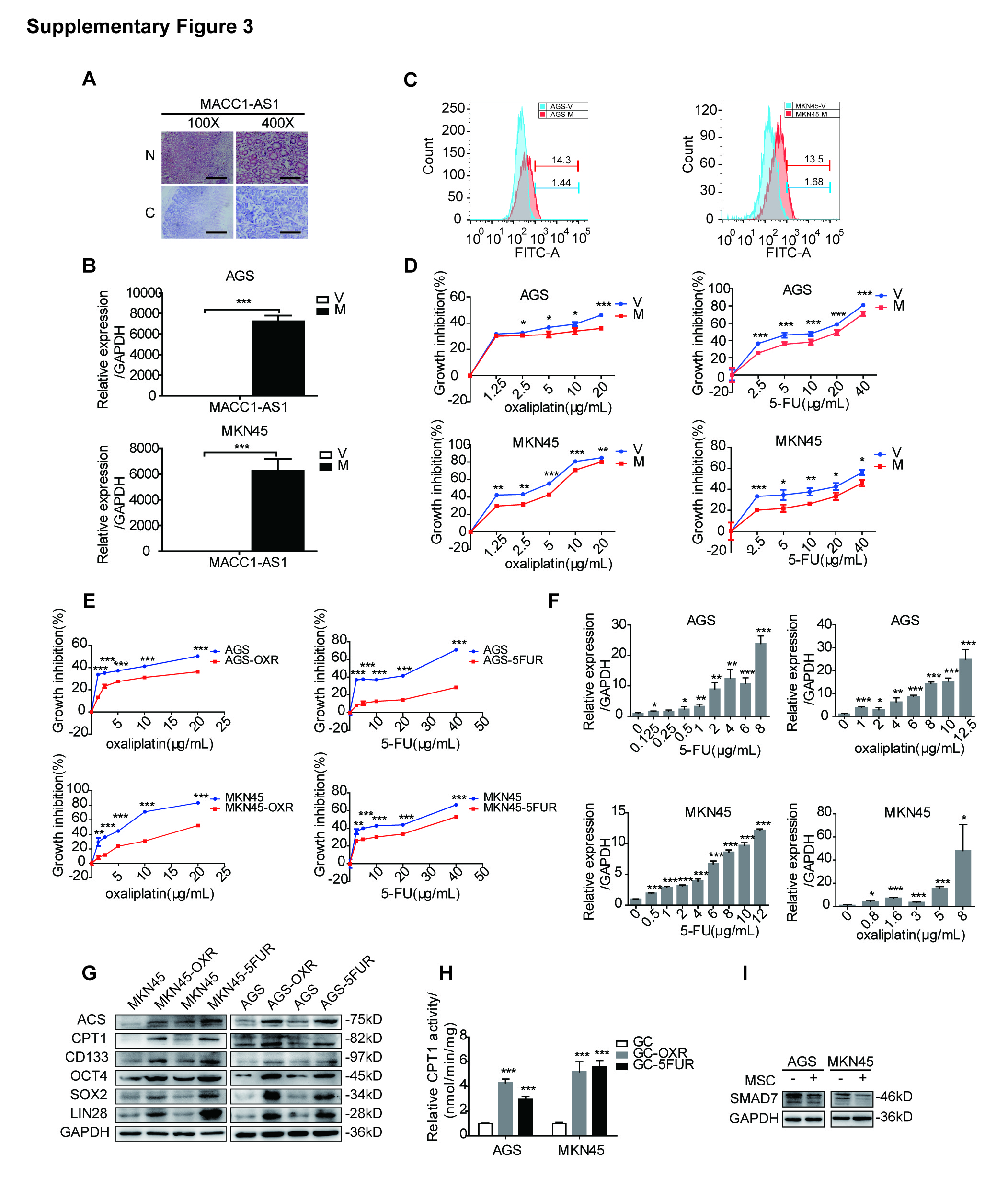

Supplement: Supplementary file 4 — Supplemental Figure 3 [file 41388_2019_747_MOESM4_ESM.jpg]

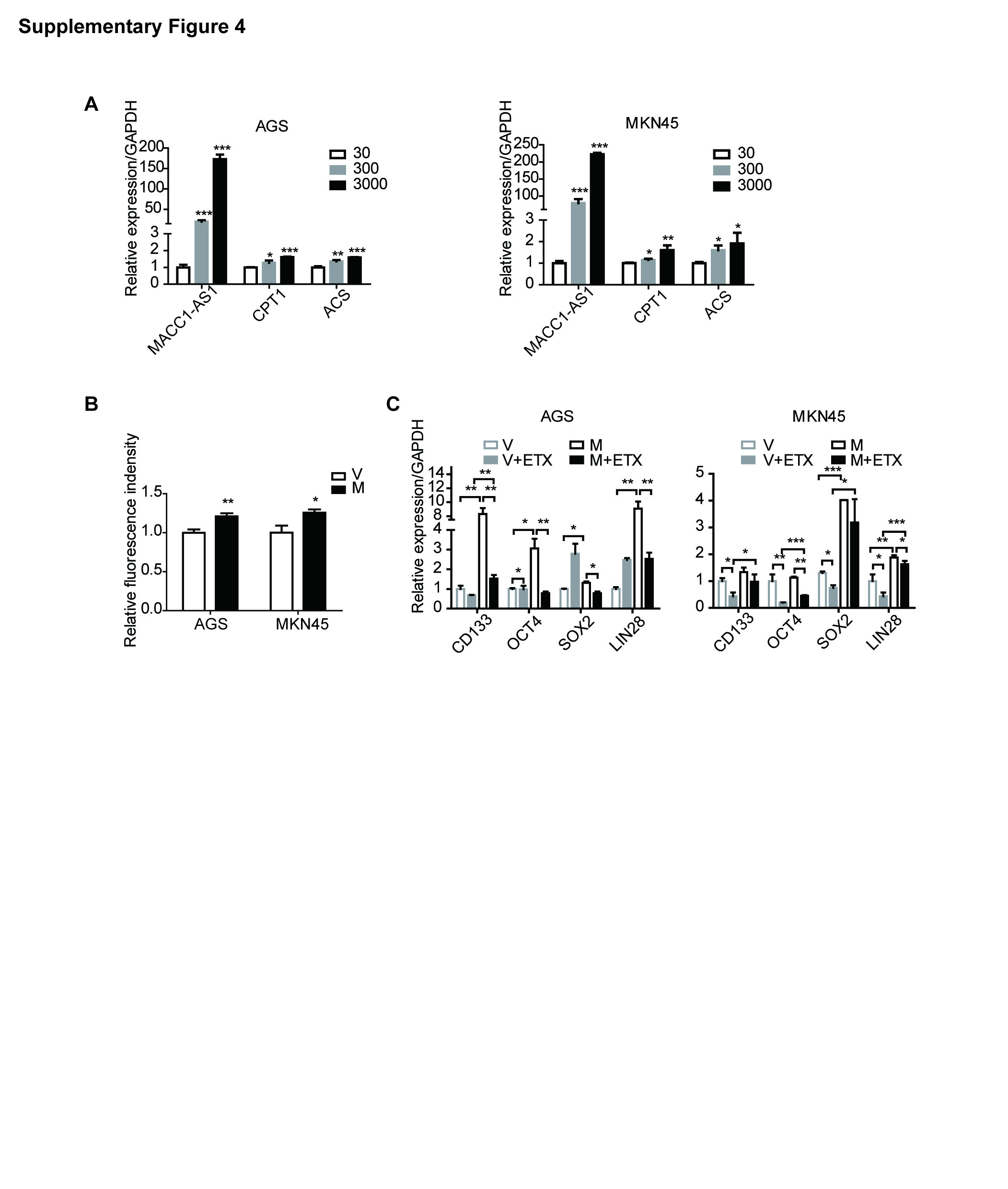

Supplement: Supplementary file 5 — Supplemental Figure 4 [file 41388_2019_747_MOESM5_ESM.jpg]

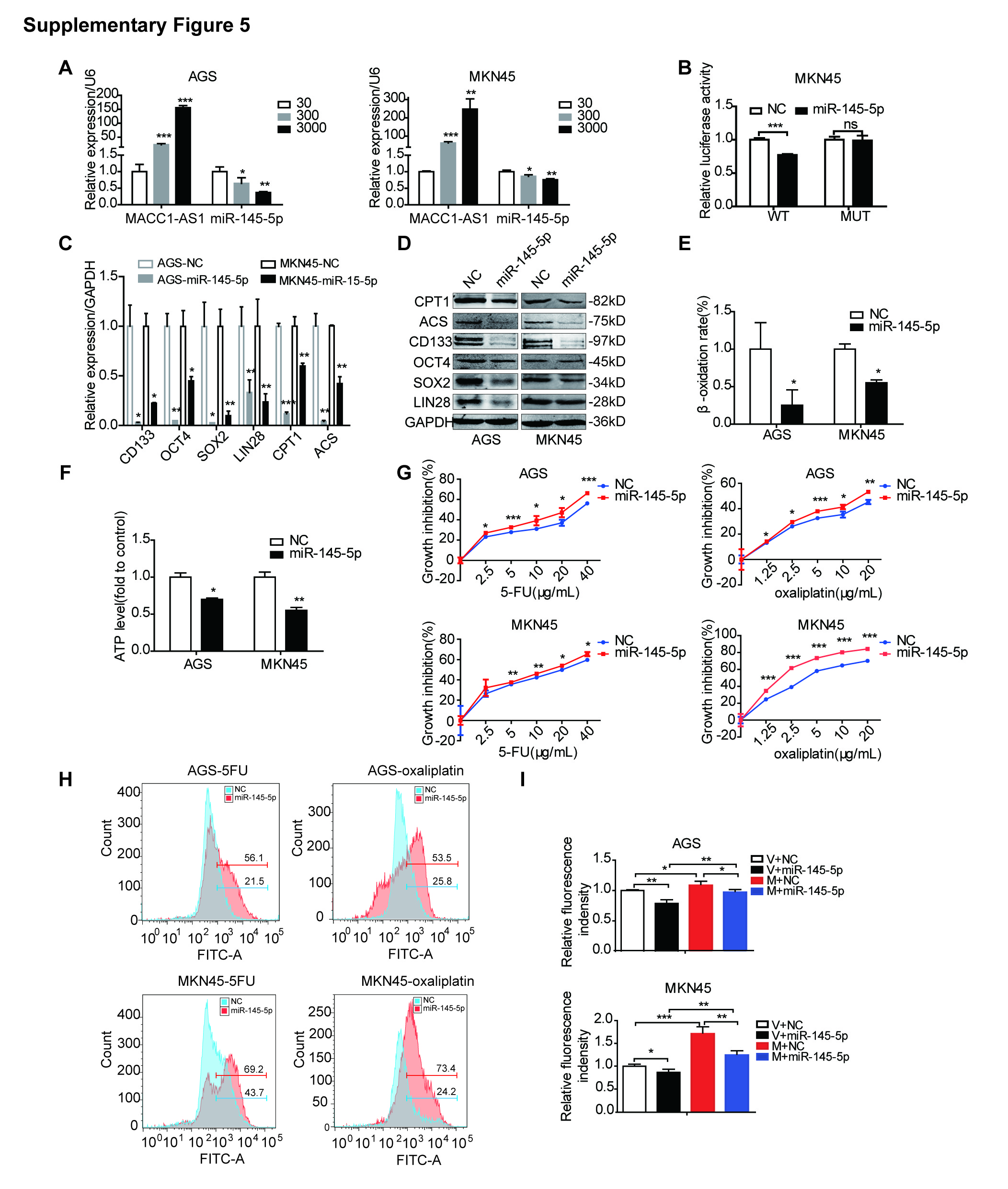

Supplement: Supplementary file 6 — Supplemental Figure 5 [file 41388_2019_747_MOESM6_ESM.jpg]
